# Where would Canadians prefer to die? Variation by situational severity, support for family obligations, and age in a national study

**DOI:** 10.1186/s12904-022-01023-1

**Published:** 2022-08-01

**Authors:** Laura M. Funk, Corey S. Mackenzie, Maria Cherba, Nicole Del Rosario, Marian Krawczyk, Andrea Rounce, Kelli Stajduhar, S. Robin Cohen

**Affiliations:** 1 Department of Sociology and Criminology, University of Manitoba, 307-183 Dafoe Road, Isbister Building, Winnipeg, Manitoba R3T2N2 Canada; 2 Department of Psychology, University of Manitoba, Winnipeg, Manitoba R3T2N2 Canada; 3 Department of Communication, University of Ottawa, Ottawa, Ontario K1N6N5 Canada; 4 School of Interdisciplinary Studies, University of Glasgow, Dumfries, Scotland DG1 4AL UK; 5 Department of Political Studies, University of Manitoba, Winnipeg, Manitoba R3T2N2 Canada; 6 School of Nursing, University of Victoria, Victoria, British Columbia V8P5C2 Canada; 7 Lady Davis Research Institute, 3755 Côte Sainte Catherine Road, Montréal, Québec H3T 1E2 Canada

**Keywords:** Dying preferences, Public policy, Perception, Place of death, Family care, Palliative care, Surveys and questionnaires, Canada

## Abstract

**Background:**

Death at home has been identified as a key quality indicator for Canadian health care systems and is often assumed to reflect the wishes of the entire Canadian public. Although research in other countries has begun to question this assumption, there is a dearth of rigorous evidence of a national scope in Canada. This study addresses this gap and extends it by exploring three factors that moderate preferences for setting of death: situational severity (entailing both symptoms and supports), perceptions of family obligation, and respondent age.

**Methods:**

Two thousand five hundred adult respondents from the general population were recruited using online panels between August 2019 and January 2020. The online survey included three vignettes, representing distinct dying scenarios which increased in severity based on symptom management alongside availability of formal and informal support. Following each vignette respondents rated their preference for each setting of death (home, acute/intensive care, palliative care unit, nursing home) for that scenario. They also provided sociodemographic information and completed a measure of beliefs about family obligations for end-of-life care.

**Results:**

Home was the clearly preferred setting only for respondents in the mild severity scenario. As the dying scenario worsened, preferences fell for home death and increased for the other options, such that in the severe scenario, most respondents preferred a palliative care or hospice setting. This pattern was particularly distinct among respondents who also were less supportive of family obligation norms, and for adults 65 years of age and older.

**Conclusions:**

Home is not universally the preferred setting for dying. The public, especially older persons and those expressing lower expectations of families in general, express greater preference for palliative care settings in situations where they might have less family or formal supports accompanied by more severe and uncontrolled symptoms. Findings suggest a) the need for public policy and health system quality indicators to reflect the nuances of public preferences, b) the need for adequate investment in hospices and palliative care settings, and c) continuing efforts to ensure that home-based formal services are available to help people manage symptoms and meet their preferences for setting of death.

**Supplementary Information:**

The online version contains supplementary material available at 10.1186/s12904-022-01023-1.

## Background

An aging population, changes in dominant causes of death and some policy and system shifts toward community-based care in Canada mean that over the last several decades hospital deaths appear to be declining, with corresponding increases in deaths in long-term residential care or at home [1]. However, the proportion of deaths at home occurring in Canada has still been relatively low compared to other countries [2]. Alongside policy emphases on aging in place and an increasing focus on cost-cutting among Western governments, Canada has been prioritizing home death/dying at home in public policy, bolstered by persistent and often unquestioned claims that this reflects peoples’ wishes and preferences [3–5].

Indeed, general population surveys and some other types of research, primarily in several Western or European countries, appear at first glance to confirm a majority preference for home death, with proportions varying between 59 and 70% [6, 7]. Some methodological concerns, however, have been raised about the evidence [7, 8], including critiques about oversimplified measurement [9], the conflation of place of care and place of death [10, 11] or of expectations and preferences [12], and a tendency to exclude respondents who express ‘no preference’ [8]. Moreover, in a few studies, greater or at least equal preference for dying in institutional settings such as hospice was reported [including Cox et al., 2013 in the UK [13]; Powell et al., 2014 in Namibia [14]; Chung et al., 2017 in Hong Kong [15]]. The use of vignette methodology may uncover more such variation in preferences [16, 17].

Alongside this emerging evidence, several UK scholars have argued that home is not the best place for all people in all circumstances [18, 19], especially given the complexity of care at the end of life [5, 20, 21]. In Canada, Hankivsky et al. [4], and in the U.S., Benson et al. [22], have identified that home death may not be desirable and/or attainable for all marginalized groups, such as those facing barriers to accessing formal services, or have, for instance, insecure housing. As such, caution is needed not to idealize dying at home nor assume people would universally orient towards the experience in the same manner.

Canadian data examining preferences for place or setting of death among the general public is still quite scarce. Wilson et al. [23] collected data in 2010 from 1203 adult respondents in the province of Alberta, which indicated that 70.8% preferred to be at home near death compared to other alternatives. A few years later, in 2013, the Canadian Hospice Palliative Care Association published the results of a national panel survey of 2976 Canadians, reporting that of those that indicated a preference for place of death, 75% expressed a preference for home [12]. However, only 52% of respondents actually expected that the majority of their care towards the end of life could be provided in their home, and around 40% of the sample expressed no preference for either place of death or place of end-of-life (EOL) care. Lastly, in an older study using a much smaller sample, Martineau, Blondeau and Godin [24] examined preferences among 138 older retired persons in the province of Québec, reporting that when faced with a scenario involving pain, 67% intend to choose hospital as a place of death; even in a scenario without pain, only 34% chose home.

A variety of research has contributed to emerging knowledge about a host of factors that may be associated with preferences to die in institutional settings in contrast to home, such as living alone/being single or widowed [in South Australia [25] and in Canada [23]]; poverty and housing precarity [in South Australia [25], the U.S. [26] and in Canada [23, 27]], or living in an urban area [in South Australia [25] and in Denmark [28]].

A body of research that is primarily qualitative in nature also suggests that considerations about symptom management and the availability of both family and formal supports (which may be affected by both social and geographic location) might affect people’s preferences for setting of death. Such research has variously indicated how practical realities (such as unavailable services), needs for medical management and symptom control, concern for safety and quality of life at home, a desire to protect family members, uncertainties associated with terminal illness, fear of dying alone, and wanting to put trust in professionals, can all potentially shift preferences towards institutional settings [11, 13, 29–33]. Indeed, the Canadian Hospice Palliative Care Association indicated that consideration of practical realities and care needs at the end of life might explain the reported discrepancies in their survey between place of death preferences and expectations among the public [12].

In 2018, Etkind et al. [34] reviewed 57 studies to synthesize factors influencing preferences for place of care, place of death, and care involvement in older adults with advanced illness or receiving palliative care. The authors identified perceived “support from and burden on family and loved ones” (p.1031) as prominent influences in this regard (and as potentially superseding personal views), as well as perceptions of what care could be provided in different settings, a desire to maintain independence or to maintain quality over quantity of life, and beliefs about future illness progression and symptoms. Moreover, preferences were not always formed or actively expressed.

In the present study, in addition to the severity of the context in which people are dying (including access to formal and informal supports, and severity of symptoms), we are interested in several other potential moderators of preferences for setting of dying. First, given that expectations of family responsibility are institutionalized within home care policies across Canada [if not EOL care policies [3]], it is important to also consider how support for generalized family care obligation norms might also shape personal preferences for setting of death. Greater attitudinal support for familialism in this regard may be associated with personal preferences for dying at home, although to our knowledge, this has not been tested in existing research. Those who express less normative support for family obligation may be less likely to want to die at home insofar as this might reflect an increased, latent concern with burdening family members. A bidirectional effect is also possible, if persons who strongly wish to be cared for and die at home manifest stronger expectations for family support to be able to do so.

Second, associations between older age and decreased preferences for dying at home have been reported in some research [12, 25, 31, 35, 36]. Etkind et al. [34] did not report an association between age and preferred place of death (but did find an association between older age and preference for home-based care towards the EOL), suggesting the research overall may be equivocal. Tentative explanations for age as a factor reducing preferences for home death include that older adults might place greater emphasis on not wanting to be a burden to their family members (who may be more likely to be older themselves, [37], especially in countries with generous public funding of alternative options [38]. Accumulated life experience may also generate increased awareness of the nuanced realities of health conditions and care needs. Cohort effects might also be operating, with older generations being generally more likely to trust in medical professionals and treatments [39]. As such, we believed that older age might be associated with stronger preferences for dying in settings other than home.

Other potential moderators of preference for place of dying might be those pertaining to peoples’ past care experiences and/or past experience with the death of a close friend/family member, although research is not definitive in this regard [6, 40]. Personality characteristics such as neuroticism may affect end of life decision making and preferences [41, 42].

In sum, given the pervasiveness of assumptions about public preferences for home death, alongside the current designation of home death as a positive indicator of health system function by the Canadian Institutes for Health Information [41], the first objective of this study was to examine public attitudes about preferences for setting of death, including dying at home or in intensive care, palliative care, or a nursing home/long-term care. Our second objective was to explore variation and nuance involved in preferences for dying – for instance, home death might be less preferred in more challenging contexts, as well as by older versus younger adults, and by respondents who express less normative support for family obligation.

## Methods

### Respondents and procedure

A sample of Canadian adults was recruited for an online study by Qualtrics^1^ between August 2019 and January 2020 (before the COVID-19 pandemic in Canada). Qualtrics used one panel partner to field the study in Canada – a panel is a group of potential respondents that can be sampled to reflect, to the greatest extent possible, the general Canadian population. Although not a purely random or nationally representative sample, panels are arguably more feasible and cost-effective alternatives to random dialing of all household phone numbers [43, 44]. To ensure sufficient numbers of participants across the lifespan, who speak both official Canadian languages, as well as men and women and those who live in both urban and rural areas, we provided Qualtrics with quotas related to age; French as first language; gender; and rural geographic location. Invitations and strategies used to recruit qualified panelists into a panel survey can vary; most panelists have access to a dashboard that lists studies they can participate in. From there they may still receive periodic invitation prompts, or they can opt-in to the survey autonomously if they so choose. Respondents are rewarded in numerous ways for opting in, including points at retail outlets and gift cards.

REB approval was received from the first author’s institution, and all methods were carried out in accordance with relevant guidelines and regulations. Respondents indicated their informed consent at the beginning of the survey. Respondents could choose to complete the survey measures described below in either official language of Canada (French and English), and on their phone or a computer (with internet access). All measures were translated from English into French, reviewed by French-language speakers, back-translated, and then checked against the original English. We “soft launched” the survey with a small sample of 200 respondents to assess data quality and response distributions prior to the full survey launch. This helped us identify slight changes to some question and response wording for accuracy. It also led us to pilot test how quickly some people could reasonably answer the survey, to derive an exclusion quality criteria in this regard of < 6 minutes. Of the 3236 people who accessed the survey and were allowed to complete it (excluding those turned away because of full quotas), 667 failed to pass quality checks and 69 were dropouts (i.e., discontinued the survey before completing it). The final sample of 2500 therefore represents a 77.2% completion rate.

### Survey

Four types of information were collected through the online survey for this analysis, presented here in order in which they were answered. First, we assessed sociodemographic information, generally following procedures and categories used by Statistics Canada.^2^ For our analysis we created three age groups representing younger (18-44), middle-aged (44-64), and older (> 65) adults.

Second, we assessed preferences for setting of dying using vignettes. Respondents were asked to imagine that they were currently dying from an illness and presented with three aspects of the situation that were modified concurrently to create mild, moderate and severe situational scenarios: the three aspects were 1) how well pain and other symptoms were managed; 2) availability and access/proximity to formal health care services; and 3) availability of informal/family support. These aspects of variation were indicated by our review of existing research findings. We pre-tested the vignettes with two focus groups of university students (*n* = 8 in each), and after further refinement, sent them to our knowledge advisory team and five caregivers or older adults in our team’s social networks, for additional assessment of the face validity of the items and question and response wording. Minor changes to wording were made as a result. The final vignettes are presented in Table 1.

**Table 1:** Survey vignettes eliciting preferences for setting of dying in three scenarios

In this section, we will show you three hypothetical scenarios in which we: (a) ask you to imagine that you are dying and have two weeks to live, and (b) ask you about your preferences for where you would want to spend the last two weeks of life.	
Vignette 1. Imagine you are currently dying from an illness, and in addition: You have only a **few symptoms** associated with the disease you are dying from, including mild pain and minor difficulties with breathing. You would rate the severity of these symptoms as **1 out of 10, where 10 is the worst possible.** You have **no difficulty coping** with these symptoms. Family and friends are **always available** to help care for and support you Health care professionals are **readily available** to visit you to attend to any health concerns you might have, even on weekends and evenings. It is also approximately 5 minutes to the closest hospital.	
Vignette 2. Imagine you are currently dying from an illness, and in addition: You have **several symptoms** associated with the disease you are dying from, including moderate pain, moderate difficulty breathing, and some difficulty walking. You would rate the severity of these symptoms as **5 out of 10, where 10 is the worst possible.** You are having **some difficulty coping** with these symptoms. Family and friends are **usually, although not always, available** to help care for and support you, and Health care professionals are **not always available** to visit you to attend to any health concerns you might have. Their availability could be delayed or limited especially on weekends and evenings. It is also approximately 30 minutes to the closest hospital.	
Vignette 3. Imagine you are currently dying from an illness, and in addition: You have **many symptoms** associated with the disease you are dying from, including severe pain, severe difficulty breathing, very limited mobility, and some problems paying attention and remembering things. You would rate the severity of these symptoms as **10 out of 10, where 10 is the worst possible**. You are having **a great deal of difficulty coping** with these symptoms. You have **no family or friends available** to help care for or support you Health care professionals are **not available** to visit you to attend to any health concerns you might have. It is also approximately 2 hours to the closest hospital.	

After each of the three vignettes was presented to respondents, they were asked to rate separately their preference for each of the four settings (with the option to add another): “In this specific situation, how much would you want to spend your last two weeks of life^3^ in the following places (where 1 is ‘not at all’ and 5 is ‘very much’): your own home or the home of a family member or friend; acute or intensive care unit; a hospice or palliative care setting; nursing home or long-term residential care; another place (please specify).” To mitigate order effects, the order of vignette presentation was randomized among respondents. Places specified as ‘other’ were recoded where appropriate, into the other categories; the remainder of responses were too small to permit inclusion in subsequent analyses. Importantly, use of the term preferences in this paper reflects the most highly rated option, rather than an explicit comparison of one option over another.

Third, we assessed support for family obligations to provide care using an adapted version of the Family Norms Scale [45] that was initially developed in French and English with a focus on caring for older frail family members. We modified the 14 items to refer to dying rather than older or frail family members (see Additional File 1). Each item is rated on a 5-point scale ranging from 1 (completely disagree) to 5 (completely agree). We reverse coded three items and then summed them to form the overall score (possible range of 14–70). Higher scores on this scale correspond to greater endorsed family obligations. In the current study, this measure had a Cronbach’s alpha of .84, indicating good internal consistency. The variable was normally distributed with a mean of 2.9 and standard deviation of .66. Splitting the scores at the median (2.93), we divided respondents into two groups indicating low versus high belief that it is the family’s responsibility to provide end-of-life care.

Fourth, we included several brief ancillary measures at the end of the survey to capture variables that might be related to end-of-life preferences. These included three questions assessing whether respondents had past experiences with: providing care to a friend/family member, the death of a close friend/family member, and being involved in decisions about life-supporting treatment. “Yes” was coded 1 and “no” was coded 0 for each question. We also asked a question about whether the person was themselves facing a life-threatening illness (1 = yes, 0 = no). Finally, we included a 2-item measure of neuroticism from the Ten Item Personality Inventory [46].

### Analyses

We examined our first research objective with a 3 (levels of situational severity: mild, moderate, and severe) by 4 (preferred settings of dying: home, acute/intensive care, palliative care, and long-term care) within-subjects repeated measures analysis of variance. Situational severity and place of death were the manipulated independent variables and ratings of preference for where participants would want to spend the last two weeks of life was the dependent variable. We followed significant effects with post-hoc pairwise comparisons using Fisher’s least significant difference (LSD) procedure. We report both statistical test information (with significant effects associated with *p* ≤ .05, two-tailed) and partial eta-squared as a measure of effect size in which small, medium, and large effects are associated with η_*p*_^2^ values of .01, .06, and .14, respectively [45]. Very few data were missing on the sociodemographic variables in Table 2 (ranging from 0 to 15 out of 2500) or on the ratings of preferences for place of death across the three severity scenarios (ranging from 8 to 15 out of 2500). We did not, therefore impute missing data. Data were analyzed using SPSS Version 27.

**Table 2:** Summary of demographic characteristics of the respondent sample

Characteristic	N (%)
**Age**	
Younger (18–44)	755 (30.2)
Middle-aged (45–64)	1002 (40.1)
Older (65+)	737 (29.5)
**Gender**	
Female	1219 (48.8)
Male	1269 (50.8)
Non-binary	12 (.5)
**First Language**	
English	1949 (77.8)
French	448 (18.0)
Other	101 (4.2)
**Citizenship Status**	
Canadian citizen	2380 (95.2)
Landed immigrant, permanent resident, refugee, other	120 (4.8)
**Relationship Status**	
Divorced/Separated	314 (12.6)
Married or common law	1330 (53.2)
Never Married/Other	732 (29.3)
Widowed	123 (4.9)
**Ethnicity**	
Arab	22 (.9)
Asian	127 (5.1)
Bi-racial or other	73 (2.9)
Black Caribbean or Black African	55 (2.2)
First Nations or Métis	47 (1.9)
Latin American	25 (1.0)
South or Southeast Asian	112 (4.5)
White	2036 (81.5)
**Religion/Belief System**	
Religious	1549 (61.5)
Non-spiritual, no religious affiliation	671 (27.2)
Spiritual, no religious affiliation	257 (10.3)
**Formal Education**	
High school diploma or less	650 (26.0)
Some college or university or higher	1850 (74.0)
**Household Income (before tax)**	
$0–$19,999	270 (10.8)
$20,000–$39,999	522 (20.9)
$40,000–$59,999	491 (19.6)
$60,000–$79,999	386 (15.4)
$80,000–$99,999	304 (12.2)
$100,000+	520 (20.8)
**Residence**	
Metropolitan area (1 million)	556 (22.2)
Large city (100,000–999,999)	803 (32.1)
Medium city (30,000–99,999)	423 (16.9)
Small city (10,000-29,999)	225 (9.0)
Town (1000-9999)	293 (11.7)
Village (300–999)	85 (3.4)
Hamlet (fewer than 300) or other	79 (3.2)
**Experiences with death and dying**	
Current life-threatening illness	131 (5.2)
Provided care for dying family member or friend	982 (39.3)
Experienced the death of a close friend or family member	2239 (89.6)
Involved in decision to stop or not start life-supporting treatment	473 (18.9)

Missing data on each of the variables in this table ranged from 0 to 15 out of 2500

Our second research question concerned moderators of dying preferences. We began by exploring correlations between our preference for dying at home variables (mild, moderate, and severe) and the variables we hypothesized might affect them. We explored both the significance of correlations, and the strength of effects where correlations of .1, .3, and .5 are associated with small, medium, and large effect sizes [47]. We selected variables for subsequent moderator analyses that had significant correlations associated with small to medium effect sizes with all three home scenario variables, resulting in two subsequent analyses in which family obligations (high vs low) and respondent age (young, middle-aged, old) were added as independent variables to the base model described above. Level of situational severity and preferred setting of death are again within-subjects factors, and family obligations and age are between-subjects repeated factors. We focus our interpretation of these moderator analyses on the significant three-way interactions between severity, setting of death, and either family obligations or age. Additional information concerning main effects and two-way interactions for these analyses (which were with only one exception significant) are available as a supplemental file.

## Results

### Respondent characteristics

Sociodemographic information for the final sample of 2500 respondents is summarized in Table 2. Respondents ranged in age from 18 to 99, with a *M*_age_ = 52.3 (*SD* = 17.4). The sample had roughly equal numbers of men and women and the majority of respondents were white, English-speaking, married, well-educated, religious and urban dwelling. Most participants had past or current experiences with death and dying. Although directly comparable census data is in most cases unavailable, our estimates suggest that the sample is roughly representative of Canadians 18+ on many sociodemographic characteristics (age, gender, income, religiosity, marital status, rural or urban residence) with slight under-representation of respondents self-identifying as ethnic minority and over-representation of highly educated respondents.

### Preferences for setting of dying across the three vignettes

We examined respondents’ preference ratings for place of dying in each scenario for the four options (i.e., home, acute/intensive care, hospice/palliative care unit, long-term care). Histogram distributions for each setting and each scenario are provided (see Additional File 2) with a score of 5 indicating highest preference.

Regarding our first research objective, there was a significant multivariate main effect of situational severity [*F*(2,2446) = 425.77, *p* < .001, η_*p*_^2^ = .26] on overall preferences for place of death, where preferences increased from mild severity (M = 2.53; SE = .02) to moderate severity (M = 2.89; SE = .02) to severe severity (M = 2.99; SE = .02). These mean scores were all significantly different from one another (all *p*s < .001). There was also a main effect of setting [*F*(3,2445) = 1187.92, *p* < .001, η_*p*_^2^ = .59] where overall preferences were highest for home (M = 3.81; SE = .02), followed by hospice/palliative care unit (M = 2.877; SE = .02), intensive/acute care (M = 2.40; .02), and lowest for LTC (M = 2.14; SE = .02). Again, all pair-wise comparisons were significant (*p*s < .001). The presence of interaction effects, however, makes these main effect findings relatively less meaningful. Importantly, the interaction between severity and setting was also significant [*F*(6,2442) = 314.02, *p* < .001, η_*p*_^2^ = .44]. The mean scores in Table 3 demonstrate that as situational severity increased, preference for dying at home decreased, whereas preferences for dying in a palliative care unit/hospice, long-term care facility, and in acute/intensive care increased. Post-hoc pairwise comparisons revealed that all comparisons were significant except at the highest severity level, where there was a non-significant difference of preference scores between home and acute/intensive care options (*p* = .325).

**Table 3:** Mean scores and standard errors for place of dying preferences across severity of the dying scenarios

Severity	Place	Severity X Place Mean (SE)	Lower – Upper 95% CI
Mild	Home	4.39 (.02)	4.34–4.43
	ICU/Acute care	1.82 (.02)	1.78–1.87
	Palliative care unit	2.16 (.03)	2.11–2.21
	LTC	1.76 (.02)	1.72–1.80
Moderate	Home	4.00 (.03)	3.96–4.06
	ICU/Acute care	2.40 (.03)	2.35–2.45
	Palliative care unit	2.95 (.03)	2.90–3.00
	LTC	2.22 (.02)	2.17–2.27
Severe	Home	3.04 (.03)	2.97–3.10
	ICU/Acute care	2.98 (.03)	2.92–3.04
	Palliative care unit	3.49 (.03)	3.43–3.55
	LTC	2.44 (.03)	2.38–2.49

*ICU* intensive care unit, *LTC* long-term residential care or nursing home, *Palliative care* hospice or palliative care unit

### Moderators of preferences for setting of dying across the three vignettes

Turning to moderators of dying preferences, correlations between preferences for dying at home were significant with small effect sizes for age, and significant with small to medium effect sizes for family obligations. None of the other hypothesized moderator variables was significantly related to preference for dying at home across the three severity scenarios (see Table 4).

**Table 4:** Correlations among study variables

	1	2	3	4	5	6	7	8	9	10	11
**1. Mild Home Scenario**	1										
**2. Moderate Home Scenario**	.33^**^	1									
**3. Severe Home Scenario**	.12^**^	.51^**^	1								
**4. Age**	−.06^**^	−.12^**^	−.13^**^	1							
**5. Gender**	.03	−.02	−.06^**^	−.14^**^	1						
**6. Family Obligations**	.07^**^	.16^**^	.20^**^	−.15^**^	−.07^**^	1					
**7. Past Care of Dying Individual**	.04^*^	.02	−.02	.12^**^	.04	.06^**^	1				
**8. Past Death of Close Family**	.05^*^	.02	−.05^*^	.20^**^	−.03	−.04	.22^**^	1			
**9. Past Decision to Stop Life Support**	.05^*^	.00	−.03	.19^**^	−.02	−.03	.28^**^	.15^**^	1		
**10. Neuroticism**	.01	.04	.05^**^	−.26^**^	.14^**^	.02	−.05^*^	−.04^*^	−.12^**^	1	
**11. Current Life-Threatening Illness**	−.02	−.02	−.01	.10^**^	−.06^**^	−.02	.04	.02	.03	−.02	1

Values indicate Pearson correlation coefficients

* *p* < .05. ** *p* < .01

**Fig. 1:**
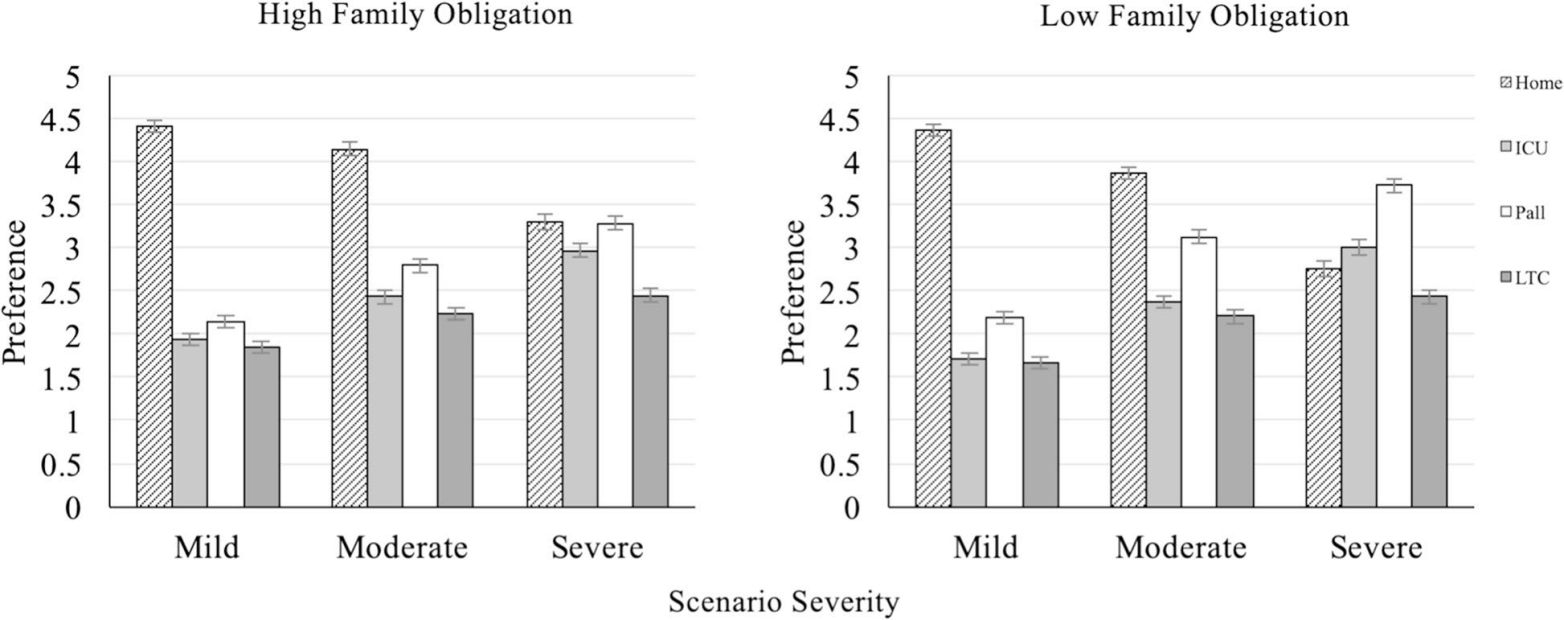
Preference for four dying options crossed by scenario severity and family obligation

With respect to the family obligation analysis, there was a significant three-way interaction between severity, setting, and family obligation [*F*(6,2440) = 9.248, *p* < .001, η_*p*_^2^ = .02]. This interaction is depicted visually in Fig. 1 and we note that non-overlapping error bars indicate significant differences. All pairwise comparisons in this Figure are significant (*p*s < .05) with two exceptions: (1) the difference between intensive/acute care and hospice/palliative care unit in the case of mild severity and high family obligations, *p* = 0.78, and (2) the difference between intensive/acute care and long-term care in the case of mild severity and low family obligations, *p* = .19. This figure shows that as the severity of the context worsens people are increasingly likely to prefer non-home dying options. In addition, for respondents with high family obligation scores, home death remains a preferred option for dying, although in the most severe situation home death is equally preferred to dying in a hospice/palliative care unit. Conversely, for respondents with low family obligation scores, home death is no longer a preferred option even in the most severe situation; both intensive/acute care and especially hospice/palliative care units are preferred options in that case.^4^

**Fig. 2:**
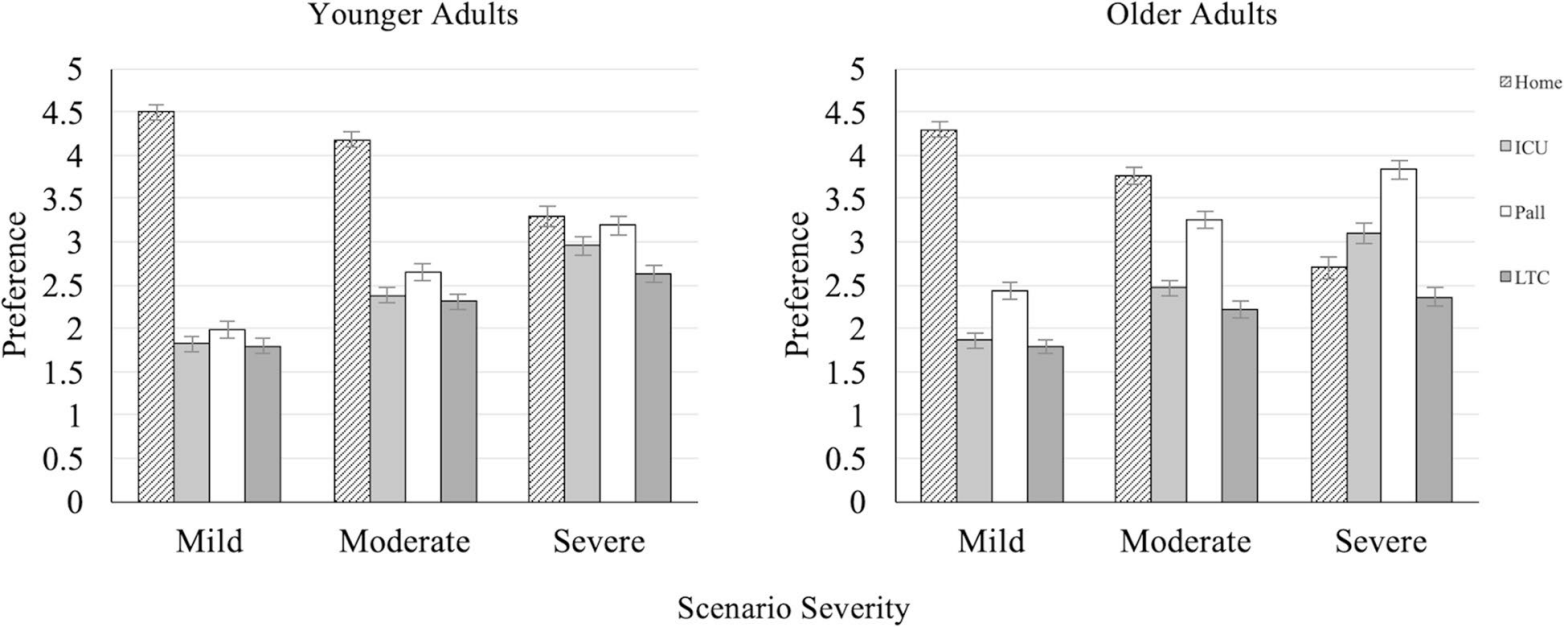
Preference for four dying options crossed by scenario severity and respondent age

With respect to the age analysis, similar to the previous analyses, as the severity of the context worsens, people increasingly prefer non-home dying options, whereas preference for home death decreases. This pattern was moderated by age as shown in Fig. 2, which omits the middle-aged group because age effects were linear such that age effects are clearest when comparing the older and younger groups. All paired comparisons between dying at home options were significant (*p*s < .05) with three exceptions: In the high family obligation group differences were not significant: (1) when severity is mild between intensive/acute care and LTC, *p* = .51; and (2) when severity is high between home and hospice/palliative care unit, *p* = .35. In the low family obligation group differences were not significant (3) when severity is mild between intensive/acute care and LTC, *p* = .09.

For younger adults, dying at home is the most preferred option even in the severe scenario, in which it is rated on par with palliative care. Conversely, for older adults, palliative care preferences approach those of home death in the moderate severity condition and by far surpass it in the severe scenario. Our oldest respondents even express a preference for dying in intensive/acute care over home in the severe scenario.

## Discussion

Confirming population-based research in other (primarily Western) countries, we can conclude from the present study that home is not universally the preferred setting for dying in this Canadian sample. The public expressed greater preference for care on a palliative care unit or hospice in situations where they might have less family or formal support, accompanied by more severe and unmanaged symptoms. This confirms emerging evidence from qualitative research about the importance of these kinds of contingencies [11, 29–32, 34]. Our findings also align with a recent Australian survey of the general older population that employed a discrete choice experiment model, and which concluded that not only was there substantial heterogeneity in preferences, but that preferences for death at home were only minimally greater than preferences for death in institutional settings [48].

In the present study, those expressing lower care expectations of families in general show greater preference for being in a palliative or hospice care setting (and to a lesser extent, acute/intensive care) in the more severe scenario we presented in the survey. Although we are unable to ascertain the causal direction of the effect, it is possible that those with lower family obligation scores were more concerned about or attuned to the potential effects of EOL caregiving on their own family members, generating a preference for care in these other two settings over home, in the most severe scenario. It may also be possible that people who express lower expectations of families in general are those who might already have less support from family (or lower relationship quality) and who therefore anticipate that it will not be possible for them to die at home in the most severe scenario.

The high family obligation group, in contrast, indicated relatively equal preference for either a palliative care unit or home in the most severe scenario. The continued high ranking of dying at home in this scenario, under these conditions, is surprising, as the conditions might highly constrain the quality and experience of dying at home. This finding might reflect either a lack of awareness about the services that can and cannot be provided in these two different settings, a sense that severe EOL symptoms are unavoidable, and/or a lack of understanding about or preparedness for what dying at home might entail [49]. There is a need for clinicians to discuss expectations in this regard with families; making sure that people understand what care can and cannot be available at home, and explain when the complexity of care needed might make it impossible to facilitate a comfortable home death. There are, however, particular challenges in this regard that may necessitate training and resources for health care providers [50–52].

Notably, even those expressing strong support for family obligations tended to have reduced support for home death in the most severe scenario, compared against the mild and moderate scenarios. Importantly, we know from some related research on eldercare in Western countries that people might strongly support both state and family care responsibility [53, 54]. The Canadian Hospice Palliative Care Association panel survey [12] likewise indicated that respondents expected EOL support from both professionals and family. Other research suggests the public has bounded, rather than limitless, expectations for their own involvement in EOL care [55]. In Canada, this bounded sense of family responsibility alongside support for state involvement has been confirmed in research examining public preferences for caring for older adult family members [45, 56, 57]. Indeed, even though high support for family care can be found among immigrant older adults [58], the authors emphasize that this does not mean that professional home care is not needed or would not be utilized among these groups.

Older persons also expressed greater preference for care within a palliative care setting or hospice (and a lesser extent acute/intensive care) as opposed to home in the most severe scenario. It is possible that the association between age and preferred setting of dying might be explained by a tendency for reduced sense of familialism in older age groups [59], or at least greater concern with family burden. However, this was not supported by our data, since older age was in fact associated with greater support for family obligations. Instead, we believe that this finding may be a cohort and/or age effect related to increased preference for medical intervention, and perhaps an increased desire to place trust in medical professionals [60]. In addition, older adults may have more experience receiving formal services like home care, and may envision that the disruption involved in their home environment towards the EOL might be too excessive. They may have more experience with how bad symptoms can get or what it feels like to not be coping well. They may also have more direct and indirect experience with what palliative care and hospitals can do for dying persons and their families - past experiences were positively and significantly correlated with age [61].

We also found that previous experience with care or death/dying was only minimally correlated with preferences, which was perhaps surprising, as previous experiences can inform our attitudes about care in particular settings. However, this lack of a strong correlation aligns with other research [23, 35].

Somewhat surprisingly, many people, especially those who are older, would want to die in an acute or intensive care setting in the severe scenario. Among individuals supporting this option, there may be a misunderstanding of palliative care settings^5^ (even fear of euthanasia), a belief that care at the end of life is still a medical issue, or a desire to ‘go down fighting’ (which may reflect socialization into an acute care mindset that infuses the rest of the health care system). This may indicate a need for public education about the limits to what care in these settings can accomplish in the last two weeks of life [62, 63].

Given that variation in preferences about dying at home does exist, we need to consider whether Canadian policies are indeed evidence-based in a way that is appropriately sensitive to the complexities of public attitudes (Cox and colleagues [13] make a similar argument in the UK). The findings from our research raise questions about the extent of public consensus on this issue [64].

Limitations of this study include its use of non-random sampling to meet quotas, which introduces the possibility of selection and non-response bias. Although the sample appears roughly comparable to the Canadian population on several key socio-demographic variables such as age, gender, and income, there was slight over-representation of White-identifying respondents and a more distinct over-representation of highly educated respondents. This may have resulted in the opinions highly educated respondents in particular being magnified. People without access to the Internet also would have been unable to complete the survey, which would include proportionately more marginalized populations and people living in more remote areas. We are also presenting sample-wide findings; in reality, there can be complex differences between provinces and territories in access to quality services and care, as well as in actual trends and patterns in places of death [65].

Even slight under-representation of cultural and visible minority groups, as well as Indigenous communities, is another concern, which meant that we could not examine differences between these groups and the majority White population. The need for further research with these populations is even more important due to trends in immigration, the lack of clear research evidence in this area, and the need for equity analyses of policy. For instance, death at home might be less preferred than in alternative settings in some immigrant and indigenous communities [4, 36, 66], yet remain the dominant preference in others [67–69]. We also know little about how culture and processes of racialization, in countries with substantial immigrant populations, shape experiences of death/dying, group variations in preferences, and the need for appropriate care [70, 71].

## Conclusions

Death at home has been identified as a key positive quality indicator for Canadian health care systems, and dying at home seems almost universally assumed to reflect the wishes of the entire Canadian public. Although some research in other countries has begun to question this assumption, this article presents the first rigorous evidence from Canada. This study contributes important information about factors that moderate preferences for setting of dying, as well as some factors that do not (e.g., neuroticism, having a life-threatening illness). Our findings illuminate some important ways in which public preferences for setting of dying depend on our perceptions of available formal and informal supports and symptom severity, and how they might, in addition, be shaped by age- or cohort-related effects as well as the extent of our normative support for family care obligations.

Resources and practices are needed that support flexibility in conditions of uncertainty and/or ambivalence. Since views/preferences are context-dependent, we need to develop supports that are responsive to changing contexts which are common at the EOL, including education about EOL as a process rather than an event that may occur across multiple locations of care, and to help family caregivers understand that if people do not die at home, this is not because they have done something wrong. Continued public and micro-level education in interactions with health care professionals is also needed about what might or might not be accomplished in particular settings of care, in the last two weeks of life.

The findings from this study further suggest a need for public policy and proxy quality indicators of a good death that reflect the complexity of public preferences, alongside nuanced public policy statements regarding home care and EOL care. In addition, the findings suggest not only that we need adequate investments in hospice and palliative care settings (and their inclusion within proxy health system indicators of good deaths), but also to ensure that home-based formal services are available to help people manage symptoms as much as possible towards the EOL. With regards to the latter, a recent report [72] has highlighted gaps in services and system vulnerabilities in this regard have been brought to light during the COVID-19 pandemic, which has simultaneously led to increased demand for palliative home care services, as more people sought to die at home during this time. Future research conducted by the team since this initial survey will ascertain whether or not public preferences have in fact shifted in this context.

## Supplementary Information

**Additional file 1.**

**Additional file 2.**

**Additional file 3.**

## Data Availability

The datasets generated and/or analysed during the current study are not yet publicly available as they are still be used for additional primary analyses and publications by the research team but are available from the corresponding author on reasonable request.

## Footnotes

1 https://www.qualtrics.com/research-services/online-sample/

2 https://www150.statcan.gc.ca/n1/en/subjects/population_and_demography

3 This was modified from a single-item measure used by Wilson et al. (2013) which asked respondents “if you were diagnosed with a terminal illness, where would you choose to spend your last two weeks of life?” [24]

4 During analysis, family obligation scores were also tested as a trichotomous variable (low, medium, high). A similar pattern of results emerged compared to family obligations as a dichotomous variable. Consequently, we present the dichotomous results for simplicity.

5 Indeed, this issue was a key focus of national consensus-building activities conducted in this country: see Palliative Care Matters: How Canada’s health system needs to change. Consensus statement of the *Palliative Care Matters* lay panel. Ottawa, Canada. November 9, 2016.
