# Supplementary material for: Where would Canadians prefer to die? Variation by situational severity, support for family obligations, and age in a national study
Source: BMC Palliat Care. 2022 Aug 1;21:139. doi: 10.1186/s12904-022-01023-1 (PMC9340714; doi:10.1186/s12904-022-01023-1)
Supplement: Supplementary file 1 — Additional file 1. [file 12904_2022_1023_MOESM1_ESM.doc]

**ADDITIONAL FILE 1**

**Family Obligations Scale Items (English-language version)**

To what extent do you agree or disagree with the following items:

Family members must help their dying relatives in return for what their relatives did for them.

Completely agree

Somewhat agree

Neither agree nor disagree

Somewhat disagree

Completely disagree

It is the duty of family members to take care of their dying relatives.

Completely agree

Somewhat agree

Neither agree nor disagree

Somewhat disagree

Completely disagree

Family members must take on all the care for their dying relatives without asking for help outside the family.

Completely agree

Somewhat agree

Neither agree nor disagree

Somewhat disagree

Completely disagree

Each family member should be free to choose whether or not to take care of their dying relatives.

Completely agree

Somewhat agree

Neither agree nor disagree

Somewhat disagree

Completely disagree

Dying persons should be able to get by without the help of their family.

Completely agree

Somewhat agree

Neither agree nor disagree

Somewhat disagree

Completely disagree

People are just as responsible for their dying parents as they are for their own children.

Completely agree

Somewhat agree

Neither agree nor disagree

Somewhat disagree

Completely disagree

It is too bad when friends and neighbours provide more support than family members to a dying person.

Completely agree

Somewhat agree

Neither agree nor disagree

Somewhat disagree

Completely disagree

Family members should take care of their dying relatives even if it interferes with their social life.

Completely agree

Somewhat agree

Neither agree nor disagree

Somewhat disagree

Completely disagree

Family members should take care of their dying relatives even if it interferes with their work or education.

Completely agree

Somewhat agree

Neither agree nor disagree

Somewhat disagree

Completely disagree

Family members should take care of their dying relatives even if it causes family conflict.

Completely agree

Somewhat agree

Neither agree nor disagree

Somewhat disagree

Completely disagree

Family members should take care of their dying relatives even if it affects their health.

Completely agree

Somewhat agree

Neither agree nor disagree

Somewhat disagree

Completely disagree

Family members should take care of their dying relatives even if it has a negative effect on other family members.

Completely agree

Somewhat agree

Neither agree nor disagree

Somewhat disagree

Completely disagree

When a dying person is unable to live at home without assistance, it is better that they live in a hospice, hospital, or nursing home.

Completely agree

Somewhat agree

Neither agree nor disagree

Somewhat disagree

Completely disagree

When a dying person is unable to live at home without assistance, it is better that they live with a family member than any other place.

Completely agree

Somewhat agree

Neither agree nor disagree

Somewhat disagree

Completely disagree

Adapted with permission from the original scale’s author [Guberman, N., Lavoie, J.-P., Fournier, M.,. Grenier, L, Gagnon, E. ,. Vézina, A. & Belleau, H (2006). “Families’ values and practices with regard to responsibility for the frail elderly: implications for aging policy” *Journal of Aging and Social Policy*.18 (3/4): 59-78.]
