# Supplementary material for: Where would Canadians prefer to die? Variation by situational severity, support for family obligations, and age in a national study
Source: BMC Palliat Care. 2022 Aug 1;21:139. doi: 10.1186/s12904-022-01023-1 (PMC9340714; doi:10.1186/s12904-022-01023-1)
Supplement: Supplementary file 2 — Additional file 2. [file 12904_2022_1023_MOESM2_ESM.docx]

**ADDITIONAL FILE 2**

**Histograms for preferred place of death for each scenario severity**

Mild Severity Moderate Severity Severe Severity

Graphs Graphs Graphs


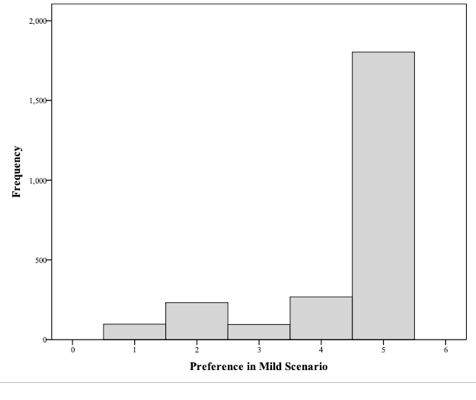

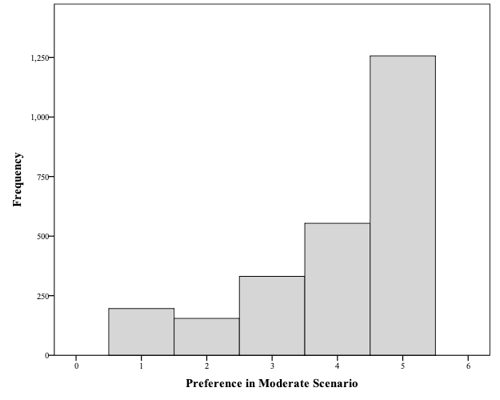

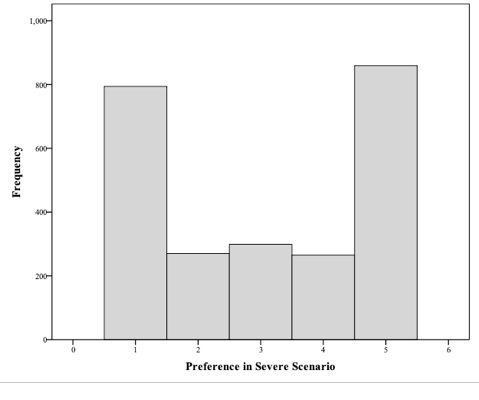


Home


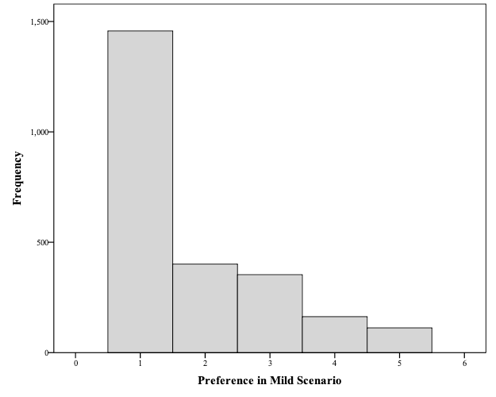

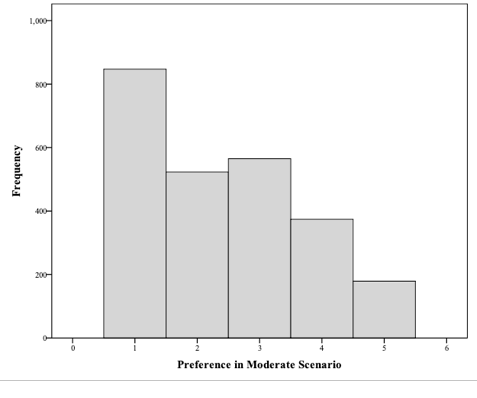

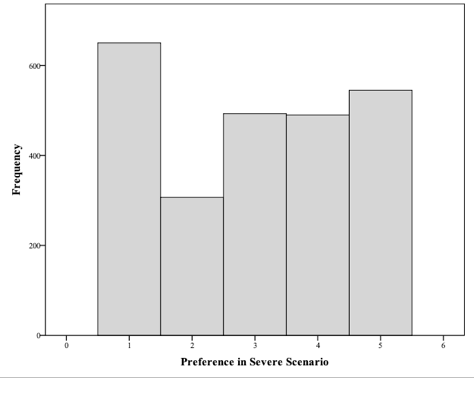


Intensive Care/ Acute Care Units


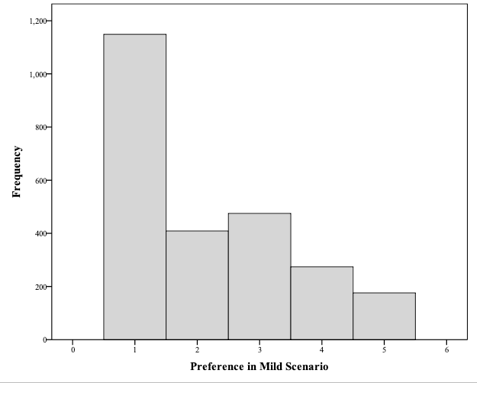

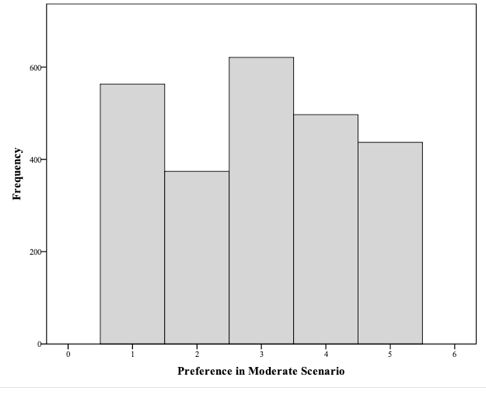

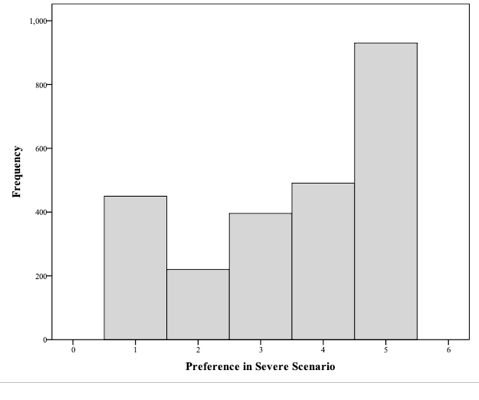


Palliative Care Units/Hospices


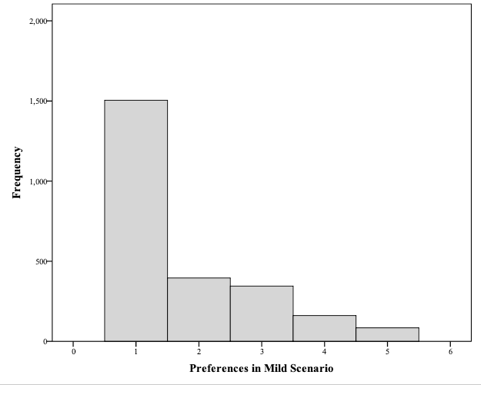

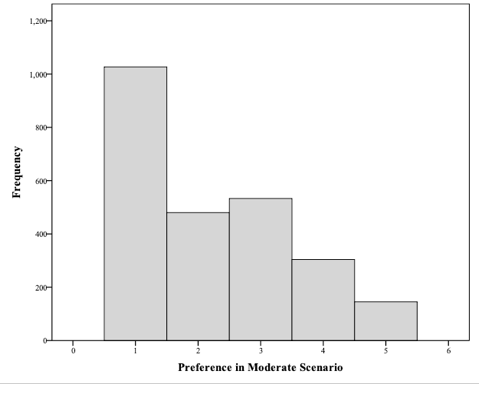

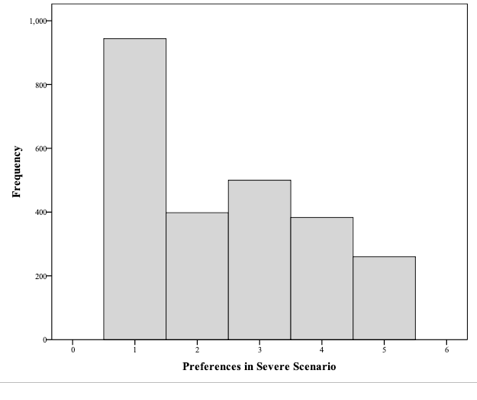
Long-Term Residential Care
