# Supplementary material for: Where would Canadians prefer to die? Variation by situational severity, support for family obligations, and age in a national study
Source: BMC Palliat Care. 2022 Aug 1;21:139. doi: 10.1186/s12904-022-01023-1 (PMC9340714; doi:10.1186/s12904-022-01023-1)
Supplement: Supplementary file 3 — Additional file 3. [file 12904_2022_1023_MOESM3_ESM.docx]

**ADDITIONAL FILE 3**

**Additional ANOVA results**

*Preferences x Familial Obligation* *across the three vignettes.*

There was a significant main effect of severity, *F*(2,2444) = 428.84, *p* < .001, ηp2 = .26, and place, *F*(3,2443) = 1191.26, *p* < .001, ηp2 = .59. Again, there was a significant interaction between severity and place, *F*(6,2440) = 323.34, *p* < .001, ηp2 = .44. There was also a significant interaction between family obligation and place, *F*(3,2443) = 33.58, *p* < .001, ηp2 = .04, and family obligation and severity, *F*(2,2444) = 5.24, *p* = .005, ηp2 = .004. There was a significant three-way interaction between severity, place, and family obligation, *F*(6,2440) = 9.248, *p* < .001, ηp2 = .02.

*Preferences x Age across the three vignettes.*

There was a significant main effect of severity, *F*(2,2439) = 411.97, *p* < .001, ηp2 = .25, and place, *F*(3,2438) = 1156.34, *p* < .001, ηp2 = .59. The interaction between severity and place remained significant, *F*(6,2435) = 310.91, *p* < .001, ηp2 = .43 and the interaction between place and age was significant, *F*(6,4878) = 29.43, *p* < .001, ηp2 = .04. The interaction between severity and age was not significant, *F*(2, 2440) = 2.21, *p* = .109, ηp2 = .002. The interaction between severity, place, and age was significant, *F*(12, 4872) = 4.06, *p* < .001, ηp2 = .01.
